# Low-Frequency Intravesical Electrical Stimulation for the Treatment of Acute Urinary Retention: A Promising Therapeutic Approach

**DOI:** 10.3389/fmed.2021.572846

**Published:** 2021-03-08

**Authors:** Tingting Cao, Bing Xie, Siyuan Yang, Jiaqi Wang, Xiao Yang, Boqiang Shen, Xueke Lin, Xiuli Sun, Jianliu Wang

**Affiliations:** ^1^Department of Obstetrics and Gynecology, Peking University People's Hospital, Beijing, China; ^2^The Key Laboratory of Female Pelvic Floor Disorders Disease of Peking University, Beijing, China; ^3^The Research Center of Female Pelvic Floor Disorders Disease of Peking University, Beijing, China; ^4^Department of Hematology and Lymphoma Research Center, Peking University Third Hospital, Beijing, China; ^5^Department of Obstetrics and Gynecology, Quanzhou First Affiliated Hospital of Fujian Medical University, Fuzhou, China

**Keywords:** rat, acute urinary retention, low-frequency transcutaneous nerve electrical stimulation, intravesical electrical stimulation, histology

## Abstract

Acute urinary retention (AUR) is a troublesome urological disease, which causes various lower urinary tract symptoms. However, only few studies explored and evaluated the effective treatments to improve AUR. We aimed to find an effective approach to cure AUR through comparing the efficacy of existing classical low-frequency transcutaneous electrical nerve stimulation (TENS) and novel intravesical electrical stimulation (IVES). A total of 24 AUR female rats were divided into 3 groups as follows: control, TENS, and IVES groups. Rats in the control group had no fake stimulation. Rats in the TENS and IVES groups underwent transcutaneous or intravesical stimulation of a symmetrical biphasic rectangular current pulse with a frequency of 35 Hz, 30 min per day, for seven consecutive days. IVES significantly reduced the actin expression in the submucosal layer but increased its expression in the detrusor layer (*p* = 0.035, *p* = 0.001). The neovascularization in the submucosal layer in the IVES group was significantly increased than in the other 2 groups (*p* = 0.006). Low-frequency IVES performed better than TENS in terms of simultaneously relieving bladder hyperactivity, accelerating epithelial recovery, and strengthening detrusor muscle. IVES may be a promising therapeutic approach for bladder dysfunction, specifically for AUR and overactive bladder in clinical practice.

## Introduction

As a severe health problem, acute urinary retention (AUR) is often caused by obstruction of lower urinary tract. In clinical practice, AUR is alleviated by relieving the obstruction, and the most common treatment is catheterization. However, it takes a long time to completely regain the bladder function after catheterization, even 39% patients changing to chronic urinary retention with or without overactive bladder (OAB) (1). Previous studies have reported that bladder overdistension of AUR induces stretch damage of the detrusor muscle, nerve, and other bladder structures, resulting in acontractile detrusor, detrusor overactivity, and low compliance bladders with/or without voiding problem (2, 3).

Low-frequency electrical stimulation (LFES) has been widely used in the urological field in recent years. The most common approach to apply LFES is transcutaneous electrical nerve stimulation (TENS). Although it is convenient and less invasive, TENS has the disadvantage of uncertain electrical energy on the bladder, and its failure rate is relatively high (4). Few studies have reported that intravesical electrical stimulation (IVES) may have direct effect on the bladder, especially on detrusor muscle, and such approach may be more effective for relieving urinary retention or neurogenic bladder dysfunction (5, 6). Streng et al. (7) have hypothesized that IVES may induce the local non-micturition muscle contractions and cause the depolarization of low-threshold mechanoreceptors in the bladder wall, which evoked sensation and subsequently a strong centrally induces detrusor contraction. Till now, there is no study focusing on the histological changes caused by IVES.

In order to verify the advantages of IVES compare with TENS and to explore its underlying mechanism, we conducted this AUR rat model study by comparing the cystometry and pathological changes between the TENS and IVES groups.

## Methods

### Animals

Briefly, 12-week-old nulliparous female Sprague-Dawley rats weighing 280–320 g (*n* = 24, Beijing Vital River Laboratory Animal Technology, China) were used in the present study. The rats were maintained under a specific pathogen-free (SPF) condition at a controlled temperature of 23°C and a 12-h light/darkness cycle, and animals were fed a balanced diet and water provided *ad libitum*. The rat model was selected because rats possess a similar anatomical structure and urinary system as humans (8). The sample size, ethical standards, criteria adopted for testing, and maintenance of the animals strictly complied with the rules established by the Chinese Laboratory Animal Requirements of Environment and Housing Facilities.

### Experimental Design

#### Establishment of Acute Urinary Retention Animal Model

The experiments were performed on rats anesthetized using 2% alpha-chloralose (with a dosage of 62.5 mg/kg) (9). To prevent possible suffering and discomfort to the rats, polyethylene (PE) tube 50 (diameters: 0.58 mm internal and 0.96 mm outside) was adapted to the dimensions of the urethra (10) and catheterized into the bladder with a length of 2.5 ~ 3 cm in order to record the intravesical pressure. Through a three-way stopcock, the catheter was attached on the one side, and a pressure transducer (MP150; Biopac, Goleta, CA, USA) and a micro-infusion pump (LD-2020II, Lande, China) were connected to the other side. The bladder capacity was defined as the infusion volume when rat urinated around the catheter, with the saline infusing into the bladder at a rate of 0.1 ml/min. Once the bladder capacity was determined through observing 3 stable micturition cycles, the bladder was infused with double amounts of bladder capacity (0.1 ml/min). Following the AUR model establishment standard by Chien et al. (11), the distal urethra was clamped by an aneurysm clip for 60 min. Then the clip was removed, and the urine could be drained out till the bladder was empty.

### Group Design

The AUR modeled rats were randomly assigned into 3 groups as follows: (1) control group (*n* = 8) without any intervention; (2) TENS treatment group (*n* = 8), and (3) IVES treatment group (*n* = 8).

#### Electrical Stimulation Electrodes

**Figure 1:**
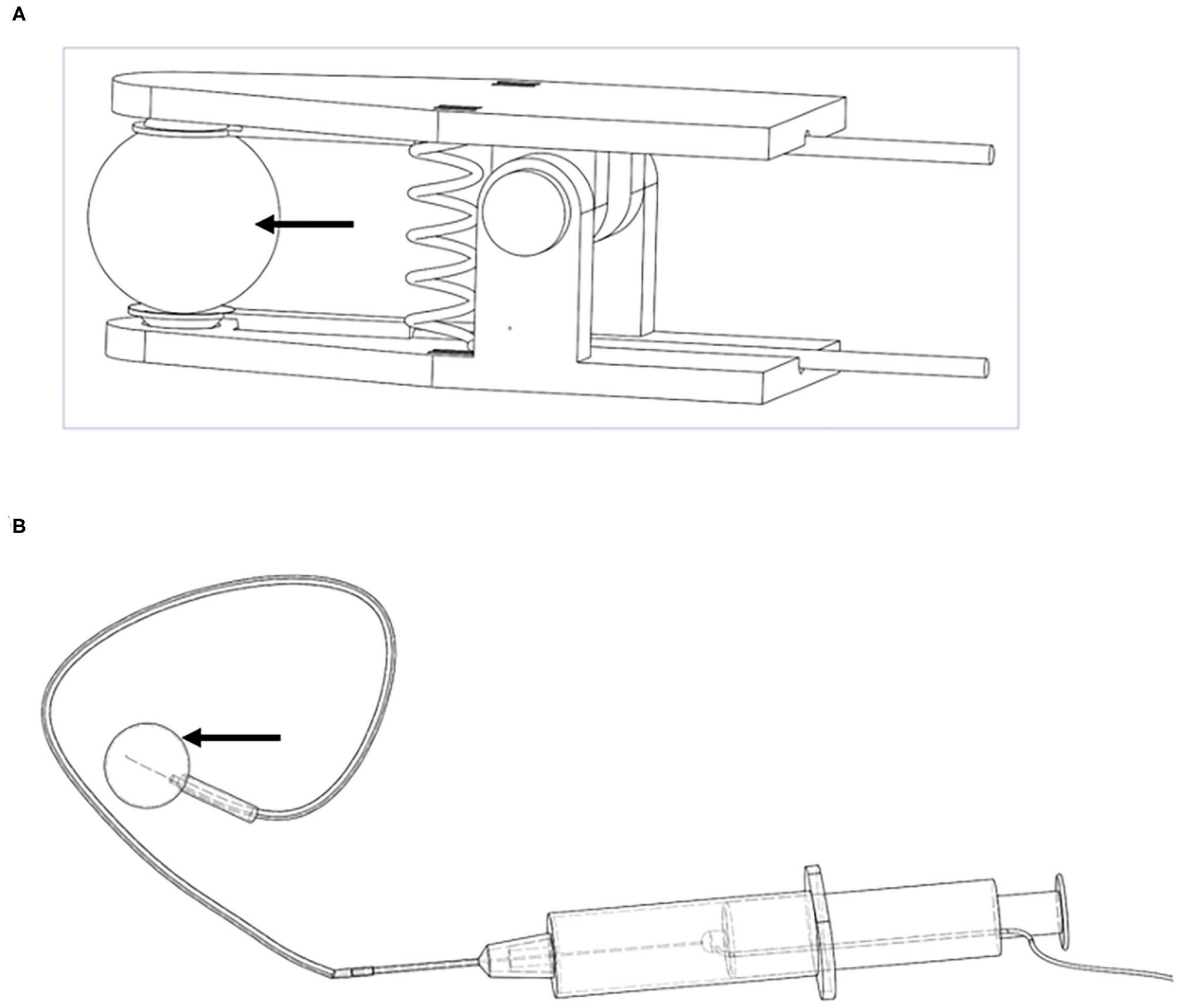
Electrical stimulation electrodes. **(A)** TENS; **(B)** IVES. Arrows denote the current area. TENS, transcutaneous electrical nerve stimulation; IVES, intravesical electrical stimulation.

The electrical stimulation electrode in the TENS group was designed as clamps (Figure 1A). The clamps were attached on the projection area of the suprapubic bladder and sacrum to form the negative and positive electrode circuits. Thus, the bladder can undergo the current loop. In the IVES group, the intravesical probe of the stimulator (diameter = 0.2 mm) was adopted and inserted to the catheterized PE tube to maintain the insulating status of the full-length urethra. The other probe was also attached on projection area of the suprapubic bladder. In order to facilitate the length of the intravesical probe into the bladder, we designed a “push electrode” by using syringe (Figure 1B). Both the intravesical and skin electrodes were made of titanium and designed by Frontier Discipline Institute of Peking University.

#### Bladder Electro-Stimulation

Before stimulation session, each anesthetized rat underwent a palpation and transcutaneous abdominal ultrasound examination to ensure the bladder was not empty, since the electrolyte was the rat urine. The skin electrodes were applied with conductive adhesive. The electrical parameters were determined according to our prospective clinical study. The two groups received the same parameters: a symmetrical biphasic rectangular current pulse with width of 200 μs, frequency of 35 Hz pulse, and an intensity of 8 mA was used until visible contraction could be witnessed by the observer without causing suffering to the animal. The time between the passage of current/muscle contraction and interruption/muscular rest (on/off) was 1-s pulse train rising edge, 4-s pulse train duration, and 1-s pulse train interval. The rats were manually placed in the supine position for the introduction of the electro-stimulator probe into the urethra and bladder. The probe was pre-lubricated and disinfected with iodophor. The rats were subjected to 30 min of electro-stimulation for one session per day in 7 consecutive days. After each session, the probes were cleaned with antiseptic soap and water and immersed in disinfectant container. During the experiment, all rats lied on an electric heating pad with a preset temperature of 37°C.

#### Cystometrogram

The cystometrograms were recorded by using AcqKnowledge 5.0 software (BIOPAC Systems, USA), which was connected with the catheter to the bladder as previously described (PE50). The recordings were collected both before and after AUR modeling. After TENS or IVES, the cystometrical evaluation was also conducted. The assessments of storage and voiding functions included the following parameters: (1) bladder hyperactivity; (2) threshold pressure (pressure at initiation of voiding contraction, P_thres_); (3) maximum intravesical pressure (P_max_); (4) compliance; (5) voiding time; and (6) post-void intravesical pressure. The P_thres_ was the pressure at the end of the sharp increase of the trace slope. Bladder hyperactivity was defined as increases in intravesical pressure during the bladder filling. The cystometrogram has rising and falling branches (12). Basal pressure before infusion was calibrated to 0 cmH_2_O. Bladder compliance was calculated by the volume change (ΔV) divided the change in intravesical pressure (ΔP, the threshold pressure-basal pressure) during saline infusion (ΔV/ΔP), which was expressed in ml/cm H_2_O. All parameters were measured 3 times, and the average value was adopted.

#### Preparation of Histological Sections

Within 30 min after the last session of electrical stimulation, the rats were sacrificed, and bladders were immediately dissected. The specimen of the anterior bladder wall near the bladder base at the level of the trigone was obtained (8- to 10-mm width, 3- to 5-mm height), and the vesical fascia was preserved to minimize the damage on the integrity of the wall. Bladder tissues of 1 mm^3^ were immersed in 3% cacodylate buffered glutaraldehyde (0.1 M, pH 7.2) for transmission electron microscopy (TEM). Other specimens were fixed in 10% formaldehyde solution, dehydrated in alcohol, diaphanized in xylene, and embedded in paraffin. In order to get a comparable histological morphology and minimize the error and bias, the tissues' direction was adjusted to the same. Subsequently, 3-μm tissue sections were cut from these paraffin blocks. All of the bladder specimens were stained using H&E staining and Masson's trichrome. Images were captured using a NanoZoomer-S360 digital slide scanner (Hamamatsu, Hamamatsu City, Japan) with a 40× lens, and the quality of the images was manually checked before application of the digital algorithm. Morphometric analyses were performed using NDP.view 2.0 software (Hamamatsu, Hamamatsu City, Japan) and ImageJ software (National Institutes of Health, Bethesda, Maryland) by 2 authors (TC and BX).

### Morphological Measurements

#### H&E Staining and Masson's Trichrome Staining

The thickness of the urothelium (200×) and detrusor muscle (80×) was analyzed by 10 random measurements. Briefly, 100 epithelium cells and spindle detrusor smooth muscle cells (DSMCs; 400×) were randomly selected to evaluate the size. To assess the results of Masson's trichrome staining, the mean proportion of collagen area was defined according to the following formula: (collagen)/(collagen + muscle). This technique was predicated on the area calculation of the smooth muscle, which was stained in red, and connective tissue, which was stained in blue.

#### Immunohistochemical Examination

The expressions of α-smooth muscle actin (α-SMA), cluster of differentiation factor 31 (CD31), and S100 protein in the bladder were assessed by immunohistochemistry. The slides were incubated with the primary antibodies against α-SMA (Abcam, 1:400), CD31 (Abcam, 1:2,000), and S100 (Abcam, 1:1,000). The proportion of α-SMA in the submucosal and detrusor muscle layers was, respectively, analyzed. The quantity and average diameter of vessel and nerve were evaluated.

#### Ultrastructure Analysis of Muscle Fibers

Semi-thin sections (1 μm thick) from each block were stained with toluidine blue and examined by light microscopy to select the most appropriate blocks for thin sectioning. Thin sections were cut and mounted on uncoated copper grids. The determination of ultrastructure was performed with Tecnai TEM (FEI, Hillsboro, USA). Myofilaments, sarcoplasmic dense bodies, sarcoplasmic reticulum (SR), and mitochondria were observed.

### Statistical Analysis

Statistical analysis was performed using SPSS version 20.0 (SPSS Inc., Chicago, IL, USA). All continuous variables were tested for normality with a Shapiro–Wilk test. The normally distributed variables were described as mean ± standard deviation (SD). The comparisons were detected by ANOVA, and Student–Newman–Keuls (SNK) *post-hoc* test was used for pairwise comparison. Categorical variables were described by percentages, and chi-square test was used to analyze the comparison. Graphs were prepared using GraphPad Prism 7.0 (GraphPad software, Inc., La Jolla, CA, USA). *p* < 0.05 was considered as statistically significant.

## Results

### Cystometry

**Figure 2:**
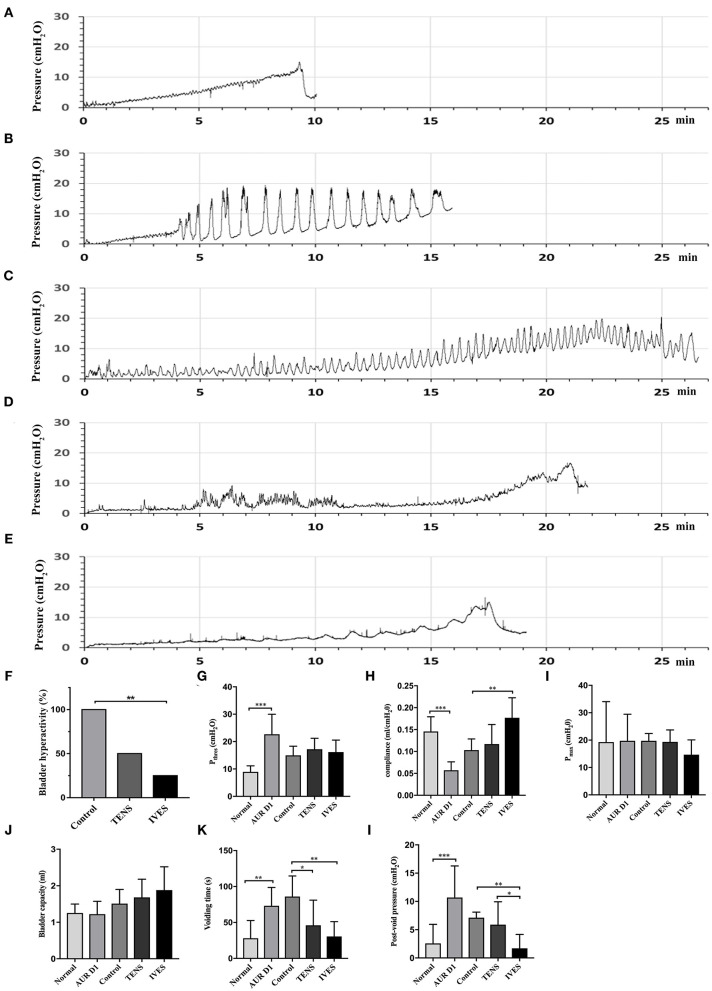
Cystometrograms. **(A)** Normal rat; **(B)** AUR rat; **(C)** control group; **(D)** low-frequency TENS group; **(E)** IVES group; and **(F–L)** quantitative analysis of cystometrical parameters. ^*^p < 0.05, ^**^p < 0.01, ^***^p < 0.001. AUR, acute urinary retention; TENS, transcutaneous electrical nerve stimulation; IVES, intravesical electrical stimulation.

During storage period, the cystometry showed that no rat had bladder hyperactivity before modeling (Figure 2A). At 24 h after the AUR model established, all rats were diagnosed with bladder hyperactivity (Figure 2B). Figures 2C,D show that bladder hyperactivity still existed in the control and TENS groups. Figure 2E show that bladder hyperactivity obviously deceased in IVES group. Compared with that in the control group, the incidence of bladder hyperactivity in the IVES group was significantly decreased (25 vs. 100%, *p* = 0.001). Although TENS reduced the incidence of bladder hyperactivity from 100 to 50%, no significant difference was observed (*p* = 0.077; Figure 2F). Once modeled, P_thres_ was significantly increased from 8.94 ± 2.22 to 22.69 ± 7.30 cmH_2_O, and the bladder compliance was decreased from 0.15 ± 0.03 to 0.06 ± 0.01 ml/cmH_2_O (*p* < 0.001). There was no significant difference in P_thres_ among control, TENS, and IVES groups (*p* > 0.05; Figure 2G). The bladder compliance was higher in the IVES group compared with the control group (0.18 ± 0.05 vs. 0.10 ± 0.03 ml/cmH_2_O, *p* = 0.004), while there was no significant difference between the TENS and control groups (*p* = 0.548; Figure 2H). There were no significant differences in terms of P_max_ and bladder capacity among the 3 groups (*p* > 0.05; Figures 2I,J). In the voiding stage, the voiding time was prolonged from 30.52 ± 20.96 to 73.18 ± 25.46 s (*p* = 0.005) once the model was established, and the post-void intravesical pressure was increased from 2.65 ± 3.4 to 10.70 ± 5.58 cmH_2_O (*p* < 0.001). Compared with the control group, TENS and IVES shortened the voiding time (*p* = 0.018; *p* = 0.002, respectively; Figure 2K). Only IVES significantly decreased the post-void intravesical pressure (*p* = 0.002; Figure 2L).

### Bladder H&E and Masson's Trichrome Staining

**Figure 3:**
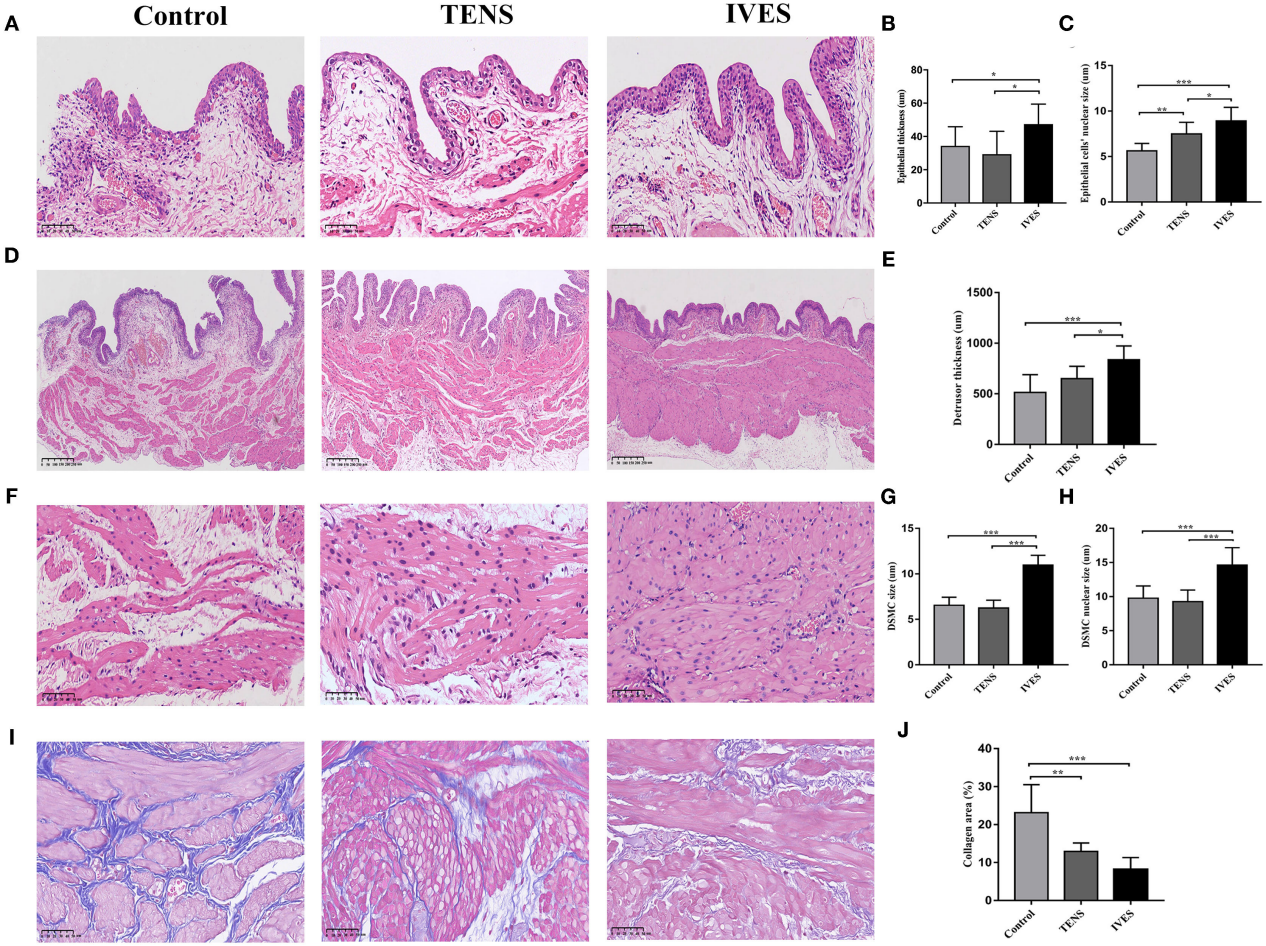
H&E staining and Masson's trichrome staining analysis. **(A–C)** Epithelium, magnification 200×. **(D,E)** Detrusor muscle layer, magnification 80×. **(F–H)** DSMC, magnification 400×. **(I,J)** Masson's trichrome staining, magnification 400×. ^*^p < 0.05, ^**^p < 0.01, ^***^p < 0.001. DSMC, detrusor smooth muscle cell.

The histological measurement showed that the epithelium was thicker in the IVES group (47.46 ± 11.98 μm) compared with the control group (34.35 ± 11.48 μm) or TENS group (29.40 ± 13.68 μm; *p* < 0.05; Figures 3A,B). The nuclear size of epithelial cells in the TENS and IVES groups was significantly larger than in the control group (*p* = 0.009, *p* < 0.001, respectively; Figure 3C), while there was significant difference between the TENS and IVES groups (7.50 ± 1.27 vs. 9.13 ± 1.47 μm, *p* = 0.034). After IVES treatment for 7 days, the detrusor layer was much thicker than in the control group or TENS group (*p* < 0.001, *p* = 0.024, respectively; Figures 3D,E). The average size of DSMC in the IVES group was larger than in the control group (10.98 ± 1.07 vs. 6.55 ± 0.88 μm, *p* < 0.001; Figures 3F,G). The nuclear size of DSMC was significantly enlarged by IVES compared with the control group (14.62 ± 2.55 vs. 9.78 ± 1.78 μm, *p* < 0.001; Figure 3H). However, the nuclear size of DSMC in the TENS group was similar with that of the control group (*p* > 0.05). The inflammatory cells in the 3 groups were not obvious. In the detrusor muscle layer, the mean proportion of collagen area in the TENS and IVES groups was significantly lower than in the control group (*p* = 0.001, *p* < 0.001, respectively; Figures 3I,J).

### Immunohistochemical Staining of α-Smooth Muscle Actin, Cluster of Differentiation Factor 31, and S100

**Figure 4:**
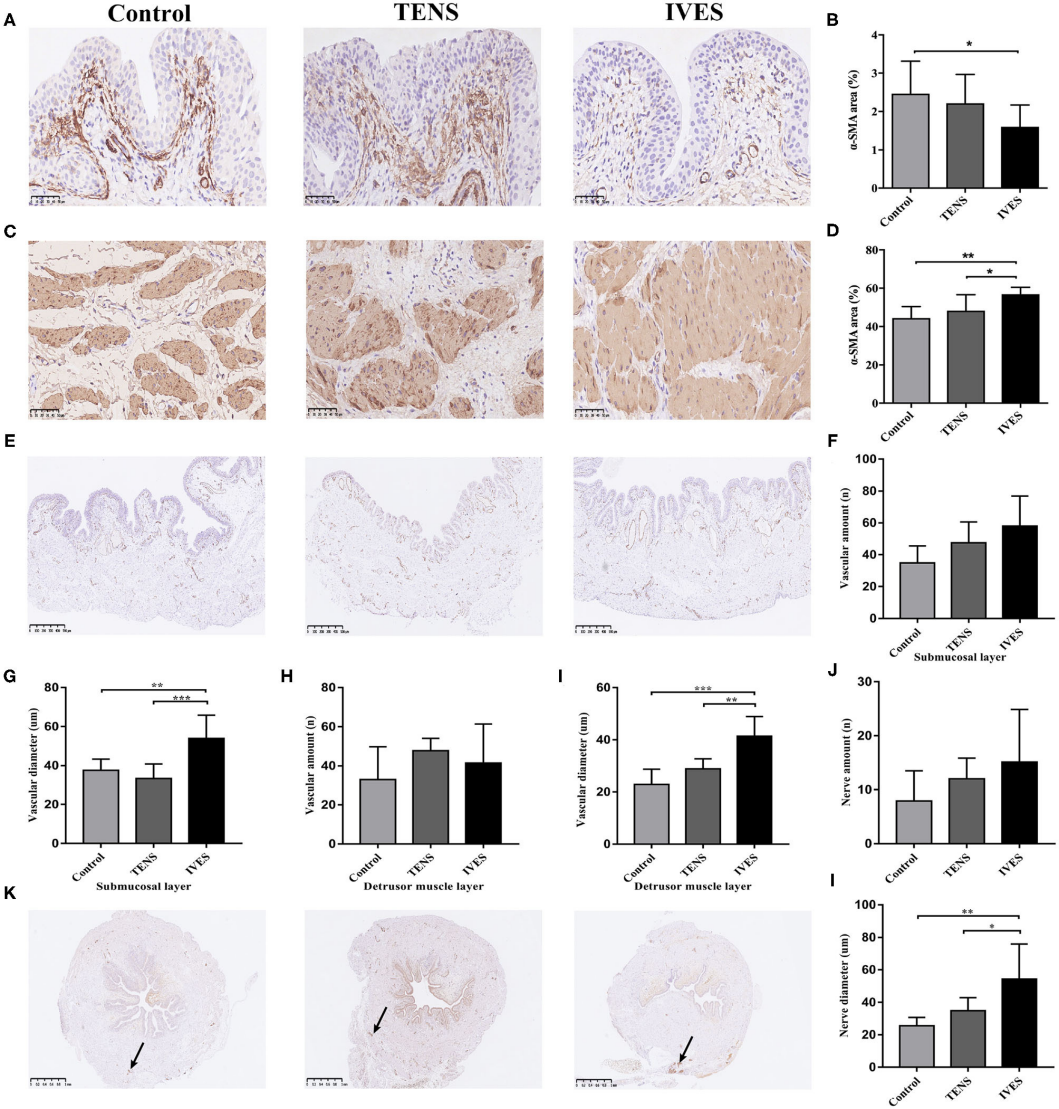
Immunohistochemical analysis. **(A,B)** α-SMA expression in the submucosal layer, magnification 400×. **(C,D)** α-SMA expression in detrusor muscle layer, magnification 400×. **(E–I)** CD31 expression, magnification 50×. **(J–L)** S100 expression, magnification 50×; arrows denote areas of positivity. ^*^p < 0.05, ^**^p < 0.01, ^***^p < 0.001. α-SMA, α-smooth muscle actin; CD31, cluster of differentiation factor 31.

Compared with the control group, IVES significantly reduced the α-SMA secretion in the submucosal layer (1.60 ± 0.57% vs. 2.47 ± 0.84%, *p* = 0.035; Figures 4A,B). The amount of blood vessels in the submucosal layer marked by anti-CD31 antibody in the IVES group was greater than in the control group (58.00 ± 18.90 vs. 34.88 ± 10.63, *p* = 0.006; Figures 4E,F). Additionally, the average diameter of blood vessels was significantly increased in the IVES group (53.91 ± 11.98 vs. 37.62 ± 5.67 μm, *p* = 0.002; Figure 4G). TENS did not change the α-SMA secretion and amount of blood vessels in the submucosal layer (*p* > 0.05). In the detrusor muscle layer, the expression of α-SMA in the IVES group was higher than in the control group (56.87 ± 3.65% vs. 44.44 ± 5.96%, *p* = 0.001; Figures 4C,D). TENS did not significantly alter the α-SMA secretion (*p* > 0.05). With respect to the amount of blood vessels in the detrusor muscle layer, the TENS group or IVES group had no significant difference compared with the control group (*p* > 0.05; Figure 4H). IVES increased the average diameter of blood vessels, and there was no difference between the TENS group and control group (*p* < 0.001, *p* = 0.064, respectively; Figure 4I). S100 staining presents nerve endings innervated closely to the serous layer. Compared with the control group, IVES significantly enlarged the average diameter of nerve (54.22 ± 21.66 vs. 25.50 ± 5.12 μm, *p* = 0.001; Figures 4K,L), while the quantity of nerve was not affected (Figure 4J). TENS had no effect on the peripheral nerve (*p* > 0.05).

### Detrusor Muscle Ultrastructure

**Figure 5:**
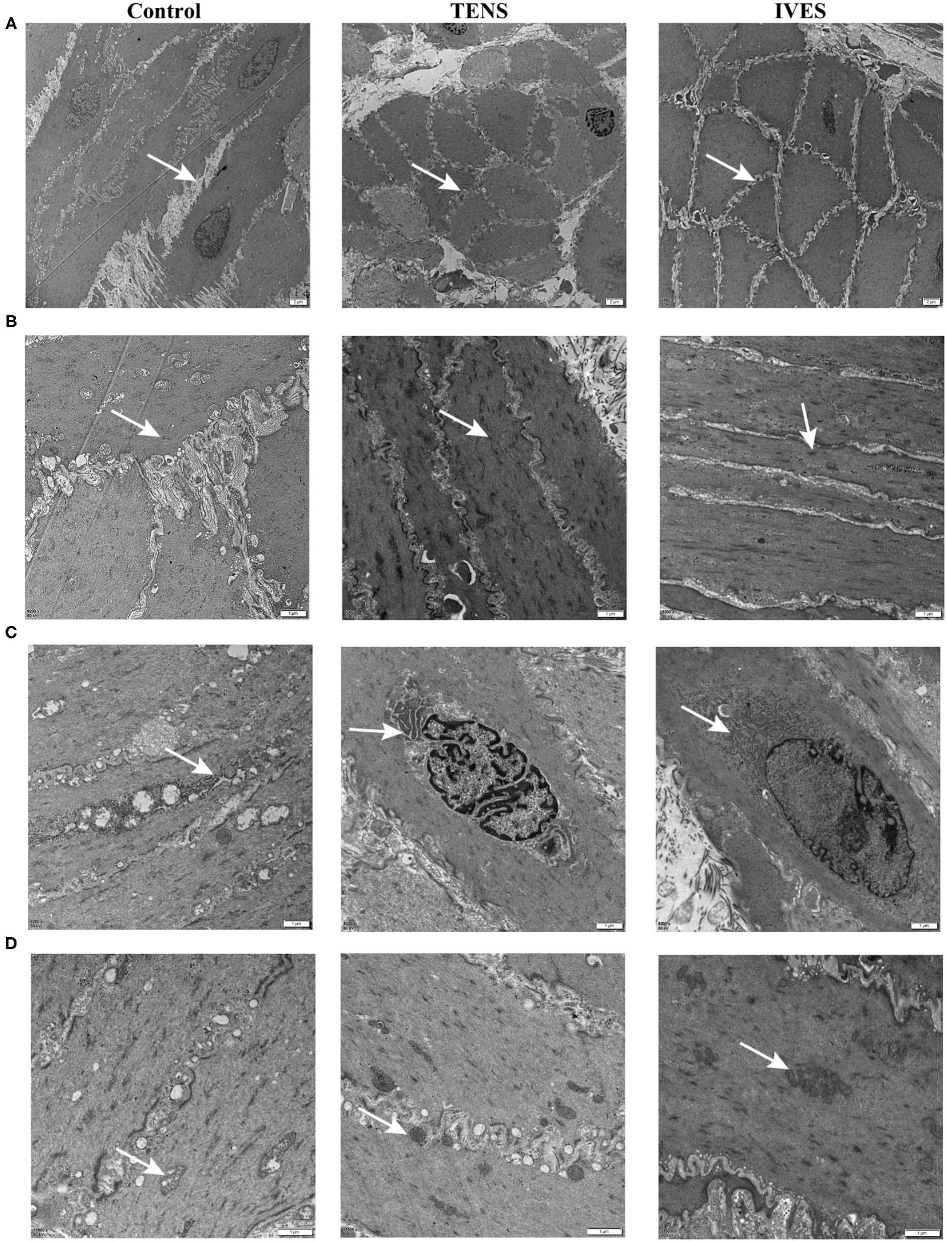
TEM of detrusor muscle ultrastructure. **(A)** DSMC arrangement, magnification 2,550×. **(B)** Dense bodies, magnification 2,550×. **(C)** SR, magnification 8,200×. **(D)** Mitochondria, magnification 11,500×. Arrows denote the ultrastructure. TEM, transmission electron microscopy; DSMC, detrusor smooth muscle cell.

At 7 days after AUR, the majority of DSMC appeared vacuolated due to dilation and disruption of cisternae of SR in the perinuclear region. Most DSMC displayed an extensive corrugation of the cell membrane with sarcoplasmic projections from 1 cell interlocking with deep infoldings of neighboring cells. After TENS or IVES treatment, myofilaments and folding of the cell membrane were regular (Figure 5A). Sarcoplasmic dense bodies were closely arranged and regularly shaped in the TENS or IVES group, while there was a non-uniform distribution in the control group (Figure 5B). Furthermore, perinuclear SR appeared greater in DSMC (Figure 5C). Grossly swollen rounded mitochondria and lysis of cristae were present in DSMC of AUR rats. However, once experienced TENS or IVES, the mitochondrial cristae were clear and arranged closely. Multiple field observations revealed that the mitochondria were more abundant in the IVES group compared with the TENS group (Figure 5D).

## Discussion

Our study revealed that AUR could affect the bladder function for more than 7 days. Cystometrical and histological results demonstrated that the effect of IVES treatment on AUR was significantly better than in the TENS. The intravesical probe material of titanium is safety and does not induce inflammation.

In cystometry study, AUR induced bladder hyperactivity and the bladder compliance became worse. We used post-void intravesical pressure and voiding time to determine the voiding function (9), which were obviously deteriorated in AUR rats. Electrical stimulation relieved bladder hyperactivity, which was similar to the research by Chen et al. (13). The cure rate of bladder hyperactivity in the IVES group was significantly higher, and the IVES group exhibited a better bladder compliance. IVES remarkably shortened the voiding time and reduced the post-void intravesical pressure. The decreased pressure can improve the bladder blood flow, and it is beneficial to the recovery of bladder function (8). However, the post-void intravesical pressure was not altered by TENS treatment. The results demonstrated that both storage and voiding functions were better improved by IVES than TENS.

Some researchers observe inflammation in the submucosa 2 weeks after the induction of AUR in the rats' bladder (3). In this study, the abscission of the epithelium was detected. The transitional cells in the bladder epithelium resisted infection. IVES enhanced the epithelial thickness and increased the layers of transitional cells, while TENS treatment and spontaneous recovery for 7 days showed no difference. AUR induces partial ischemia, and AUR-associated bladder inflammation is attributed to reactive oxygen species caused by ischemia–reperfusion (8, 14). Saito et al. have demonstrated that AUR significantly increases the vesical pressure and decreases the blood flow (8). CD31 immunostaining proved that IVES increased the density of blood vessels in the submucosa, particularly in the great vessels, enhancing the output of blood flow for epithelial recovery. Some groups have highlighted the importance of the suburothelial layers in terms of the urinary sensory system, especially suburothelial myofibroblasts (15). The number of submucosal myofibroblasts is related to bladder hypersensitivity. α-SMA is used as a marker for a subset of activated fibrogenic cells, which are regarded as important effector cells of tissue fibrogenesis (16). Through reducing the α-SMA expression in the submucosa, IVES largely decreased the incidence of bladder hyperactivity.

Prolonged bladder overdistension has been deemed to induce stretch damage of the detrusor, denervation, and reduced contractility (17). Comiter et al. have reported that the sacral nerve stimulation in rats with urinary retention can prevent functional and structural changes in the DSMC without affecting the detrusor contraction (18). We presented that the detrusor in AUR rats was thinner, and the DSMC showed atrophy. There are important filaments (myosin, actin) inside the DSMC, which are responsible for cell contraction and maintenance of normal cell shape. The ratio of actin to myosin filaments is 15:1, and the actin filaments play a vital role in contraction (19). α-SMA constitutes the contraction domain (20). TENS did not alter the α-SMA expression. IVES significantly increased the expression of α-SMA in the detrusor layer, strengthening the detrusor. These changes could improve the muscular function, thereby promoting muscle hypertrophy and conferring a better resistance to fatigue (21). Denervation and inflammation alter the expression of smooth muscle extracellular matrix, such as collagen content. Collagen in the bladder wall is present between muscle fascicles, which can decrease the bladder compliance. The Masson staining showed that TENS or IVES treatment reduced the collagen secretion, leading to enhanced compliance. The possible reason for better improvement in the IVES group could be that urine acted as conductive fluid, and the current was uniformly distributed.

It is known that electrical stimulation acts directly not only on muscle fibers but also on nerve reflexes (22). Activation of the inhibitory sympathetic neurons and inhibition of parasympathetic excitatory neurons that go to the bladder may play a role in the action mechanism of the electrical stimulation in the treatment of bladder hyperactivity (23). Erlandson et al. have highlighted that the reflexogenic pathways are described in intravaginal electrical stimulation in animals (24). Another study has also proposed that the pudendal nerve can be activated (25). In our study, nerve staining of S100 demonstrated that IVES (35 Hz) treatment could enlarge the peripheral nerve fibers' diameter, which might increase nerve conduction velocity, but not regenerate nerve. TENS treatment had no influence on nerve. This finding was consistent with previous studies that high-frequency electrical stimulation (HFES) contributes to a better nerve regeneration potential than LFES (26, 27).

Holm et al. have pointed out that no significant ultrastructural features are noted in the detrusor muscle of 13 AUR patients (28). One study focusing on rabbit bladder has presented the microstructural damage in response to partial outlet obstruction and correlated these observations with the contractile dysfunction (29). Andersson and Arner have presented that adenosine triphosphate (ATP) is a major contributor to detrusor contraction in rats (19). Our TEM results showed that mitochondria, where ATP is produced, were still disrupted in AUR rats after 7 days of recovery. Therefore, bladder overdistension followed by oxidative damage reduced the tissue ATP content, leading to detrusor dysfunction. Electrical stimulation accelerated the process of mitochondrial repair, especially in IVES. The synthesis factory of α-SMA, SR, was larger in the TENS or IVES group. Obviously, electrical stimulation increased the density of dense bodies to improve the bladder function. Dense bodies are important components in the smooth muscle cell, which are found in the cytoskeleton, suggesting that they can coordinate tensions from both contractile machinery and cytoskeleton. The actin thin filaments attach to dense bodies in the cell and provide fixed points for cell shortening (30).

The major strength of our study was the tetanic intravesical probe, and we used cystometrical and histological analyses to ensure its effect. However, there are also some limitations. The AUR rat model was not completely equal to the AUR patients, and much more animal models with lower urinary tract symptoms should be employed to confirm the efficacy of IVES. A sham group with catheter in the bladder and no stimulation would be added in further studies, which would control for histological changes of the bladder that may be secondary to the presence of the catheter and not by stimulation. There was an increase in the thickness of the detrusor in the IVES group, which might not be a desired event for patients with lower urinary tract dysfunction; thus, the high-resolution ultrasound will be added to observe and compare the *in vivo* thickness of the bladder wall in our further study. Longer observation duration might be necessary.

## Conclusions

In conclusion, low-frequency IVES performed better than TENS in treating AUR. IVES relieved the bladder hyperactivity by reducing the submucosal fibroblasts, accelerated the epithelial recovery through increasing transitional cell layer and vascular amount, and strengthened the detrusor muscle by promoting α-SMA expression. As catheterization is still an adjuvant therapy for AUR patients, it is technically possible to place intravesical probe into the bladder through catheter without additional invasive intervention. Collectively, IVES could be a promising treatment for bladder dysfunction in clinical practice.

## Data Availability Statement

The original contributions presented in the study are included in the article/supplementary material, further inquiries can be directed to the corresponding author/s.

## Ethics Statement

The animal study was reviewed and approved by the Institutional Review Board (IRB) of the Peking University People's Hospital, Beijing, China (No. 2019PHE020).

## Author Contributions

TC, BX, and SY designed, performed, and interpreted the experiments. JiaqW offered the experimental materials. XY, BS, and XL participated in the experiments. TC and BX interpreted the data and edited the manuscript. XS and JianW finalized the manuscript. All authors contributed to the article and approved the submitted version.

## Conflict of Interest

The authors declare that the research was conducted in the absence of any commercial or financial relationships that could be construed as a potential conflict of interest.
